# Content Matters, a Qualitative Analysis of Verbal Hallucinations

**DOI:** 10.3389/fpsyg.2018.01958

**Published:** 2018-10-26

**Authors:** Nienke Moernaut, Stijn Vanheule, Jasper Feyaerts

**Affiliations:** Department of Psychoanalysis and Clinical Consulting, Ghent University, Ghent, Belgium

**Keywords:** auditory verbal hallucinations, AVHs, psychoanalysis, interviews, thematic analysis, narrative analysis, psychosis, schizophrenia

## Abstract

Auditory verbal hallucinations have traditionally especially been researched from a form-based approach, with content getting much less attention. In this article, we argue for the importance of looking at content to get a fuller understanding of the hallucinatory experience. Guided by Lacanian psychoanalysis, we conducted a thematic and a narrative analysis on interviews with 10 schizophrenic patients about their hallucinations. We discerned five themes in the data, which were based on Lacanian theory and had to do with existential questions: *parenthood and authority, sexuality and relationships, gender identity, life in the light of death*, and *what does the other want?* Furthermore, we added a theme for unclassified content. Narratively, we found that participants constructed a story of four steps about their hallucinatory experiences. These steps were *disturbing events in the past posing an existential question, triggering event, period of confusion*, and *hearing voices that allude to existential themes*. Participants succeed in different degrees in integrating their hallucinatory experiences in their own life history. These stories can be situated on a continuum by making use of three prototypical narrating styles: the *meta-delusional, delusional*, and *chaotic narrative type.* Overall, our analysis shows that hallucinations can both be thematically and narratively organized, by making use of a theoretical framework like Lacanian psychoanalysis. Our research demonstrates that hallucinatory contents are not random but are about existential issues imbedded in a life narrative. Future research would benefit of integrating content and form-based approaches.

## Introduction

Hallucinations are one of the key symptoms of schizophrenia (APA, 2013), with auditory verbal hallucinations (AVHs) being the most common (Baethge et al., 2005). Most contemporary AVH theories study AVHs in terms of formal disturbances. Several cognitive models conceptualize AVHs as errors in source monitoring (Waters et al., 2012). This is shown in Frith’s theory who, among others, frames hallucinations as resulting from a loss of agency: patients no longer recognize thoughts or (inner) speech as coming from themselves. As a result, they experience thoughts as voices coming from without (Farrer and Frith, 2002). Phenomenological approaches in turn conceptualize psychosis as a disorder of self-experience, with hallucinations being an expression of an altered self-state: self-alienation creates a loss of “mineness” regarding personal experiences. Thoughts and inner speech are no longer experienced as belonging to the self, leading the way for experiencing them as hallucinations (e.g., Henriksen et al., 2015). Though Karl Jaspers in 1962 argued that there is a link between patients’ life-experiences and the content of AVHs (McCarthy-Jones, 2012), research focusing on the content of AVHs is scarce.

However, integrative models with attention to both form and content were developed in recent years as a response to challenges to exclusively form-based models, i.e., the selectivity problem (not all thoughts are experienced as hallucinations) and the specificity problem (voices often address the same, non-neutral content; Gallagher, 2004, 2005). Gallagher, for example, proposed an extension of cognitive models, according to which subconscious emotions trigger the processes responsible for the feeling of alienation and thus hallucinations. These emotions are an anticipatory reaction to the content that will be addressed in thoughts or (inner) speech. The idea that emotions can trigger hallucinations and even have an effect on the content of hallucinations can also be seen in the work of Garety et al. (2001) and Freeman and Garety (2003). From within phenomenology, Fuchs argues that in psychosis the as-if function (the ability to metaphorize, to distinguish reality from imaginary) is lost. Within this view, hallucinations are the result of thoughts about what others might say (Fuchs, 2017). These approaches are thus compatible with the view that hallucinations bear a link with personal life; however, more detailed information about voice content remains absent.

Although relatively scarce, research with a specific focus on voice content exists. However, the majority of such research fails to grasp the specific themes which are addressed by voices, as it remains limited to dividing voice content into vague categories. For example, the McCarthy-Jones et al. (2014) study uses rather general categories such as “persecutory, obscene, insulting… helpful, guiding, affirming…” Whereas qualitative research contains a more in-depth exploration of phenomena, in the case of hallucinations this has resulted primarily in insight in, for example, identity and power of voices (Holt and Tickle, 2013), but not necessarily in content of voices. Beavan and Read (2010) tackle the issue of content, stating that voice content is meaningfully related to personal experiences, but all the same their themes fail to address content-wise specificities of personal experiences and instead focus on the kind of messages voices communicate (e.g., information, criticism, etc.). Overall, until now voice content has been given minimal focus within the scientific literature. One of the exceptions can be seen in the trauma-literature on hallucinations. From within this tradition, voices are highly attributed to and linked with past experiences, especially child-abuse (e.g., Strand and Tidefors, 2012). However, a sole attribution to trauma seems to be a too narrow account to understand this complex phenomenon.

A comprehensive framework to approach the content of hallucinations is seen in Lacanian psychoanalysis. While also paying attention to formal disturbances, Lacanian psychoanalysis pays much attention to the content as well. The psychoanalytic interest in the content of psychotic experiences dates back to Freud, who in 1911 published his famous Schreber study (Freud et al., 2006). In this study, Freud sets out to understand the internal logic of psychosis, as such paying attention to the themes that come up in Schreber’s psychosis, like transforming into a woman, becoming Gods wife and giving birth to a new human race. Following on, Lacan (1955–1956, 1959) elaborated further on these foundations, as such building his own theory regarding psychosis. According to Lacan, hallucinations come to the fore when a person is challenged to position himself with respect to vital questions pertaining his own existence (who am I?) and the intentionality of the other (what do you want?), but fails to do so. Lacan denotes these questions as “real themes” – real in the sense that they, to a certain extent, escape from our comprehension. More specifically, these questions are expressed with issues regarding authority and parenthood, sexuality in relation to love and procreation, gender identity, and the meaning of life in the light of death. As these questions touch upon our essence as human beings, we will further refer to these as “existential questions.” Whereas in conventional neurotic functioning these questions can be tackled with the use of tradition, in psychosis such general tools do not function, denoted by Lacan as the foreclosure of the Name-of-the-Father. Thus, the subject is forced to devise its own original answers toward these questions. When these answers fail or are absent, a confrontation with existential questions can trigger a psychotic break. This starts with a period of confusion, followed by more clear-cut psychotic experiences, among which hallucinated voices that allude to those specific questions (Lacan, 1959; Vanheule, 2011, 2017; Leader, 2012).

In this study, we make use of the Lacanian framework to explore the content of hallucinations. As previously mentioned, in-depth research on hallucinatory content is largely missing. By making use of a theoretical framework to guide our investigation, we aim to provide a better understanding of voice content. More precisely, this research consists of a qualitative analysis of interviews with patients diagnosed with schizophrenia about their AVHs and falls into two components. The first part of our study consists of a thematic analysis, which searches to map the themes that arise when people talk about their hallucinations. Starting from our Lacanian framework, we explore whether voice content can indeed be understood as allusions on existential questions people struggle with. The second part of our study consists of a narrative analysis that aims to explore stories people construct about their hallucinatory experiences. More specifically, we will look at the different steps that can be discerned from participants’ narratives. Thereby we will start from the sequence of events put forward by Lacan: (1) triggering event, which confronts the person with an unanswerable existential question, (2) period of confusion, and (3) voices alluding to existential questions. Moreover, we also focus on how this story is disclosed and how the different elements are accounted for.

## Materials and Methods

The participants in this study were recruited in the context of a bigger project concerning the experience of psychosis. The ethics committee of Ghent University Hospital approved this project. For this project, participants were interviewed in general about their hallucinatory experiences. The interviews were semi-structured and guided by overall principles of qualitative interviewing (Smith and Osborn, 2008; Kvale and Brinkmann, 2015). The interviews always started with a question about the hospitalization history of a participant and from there on elaborated further on psychotic experiences that were brought up by the participant. The interviewer explored the nature of these experiences, with a focus on both form and content, with questions as for example: *“What do the voices say exactly? How do these voices differ from voices coming from other people? How do you react when the voices are talking like you that way?.”* Thereby, special attention was paid to the content of hallucinations, however, without specifically soliciting certain themes. As such, we aimed at getting a broad and spontaneous narrative about psychotic experiences and the role these played in participants’ lives, now and in the past. For the purpose of this study, we limited our analyses to parts of the interviews pertaining to the themes present in the hallucinations, possible triggering life events, and important related themes. The interviews and analyses were conducted in Dutch. The illustrative fragments in the results section are translations by the first author, and checked by the second author.

The participants comprised 10 patients with a DSM-5 (APA, 2013) diagnosis of schizophrenia with hallucinations and were all Flemish-speaking Belgians (for more information, see Table 1). Eight of them were recruited via the hospital where they were treated. The two others sought contact with the research group themselves, as they felt the need to narrate about their experiences. Number and length of interviews differ between participants, as not all were equally able and/or willing to give a detailed contextualized account of their experiences. As a result, more material is available for some participants than compared to others. However, as less material reflects the inability to elaborate further on their experiences, more or longer interviews would not have solved this issue. The (in)ability to narrate experiences is further addressed in the narrative analysis section.

**Table 1:** Participants’ and interviews’ characteristics.

Participant^∗^	Sex	Age	# Interviews	Interview duration^∗∗^
Bernie	M	34	2	73/43 min
Drew	M	35	3	60/44/46 min
Gudrun	F	62	2	40/55 min
Howard	M	57	2	50/44 min
Joe	M	65	2	33/39 min
Kenny	M	32	1	63 min
Matts	M	36	3	72/67/57 min
Roger	M	55	2	66/74 min
Sophie	F	19	1	39 min
Wesley	M	Unknown	1	42 min

*^∗^Names are pseudonyms to protect anonymity of participants.*

*^∗∗^Interview duration rounded up to a minute.*

To answer our first research question, we used thematic analysis, as outlined by Braun and Clarke (2006) and with support of NVivo 11 software. At first, the interviews were transcribed verbatim. Next, the first author familiarized herself with the data, via several readings of the transcripts. Subsequently, initial coding, focused on type of content, ensued. The initial coding was conducted in a bottom-up fashion, i.e., without making use of the theoretical framework, which led to 69 initial codes. In a next phase, we reorganized these initial codes into higher order themes. These themes were derived from Lacanian theory and thus consisted of the existential questions which were put forward by Lacan: *parenthood and authority, sexuality and relationships, gender identity, life in the light of death*, and *what does the other want?* We also added a theme *unclassified content* for data-fragments that did not fit into one of the former themes.

As we searched to link voice content to confrontations with existential themes in the life history of participants, we divided all existential themes into subcategories, depending on where the themes occurred: in the hallucination content, in interview comments on the hallucination, in delusional accounts, or in commentaries on life history. The final step of the thematic analysis was the write-up in Section “Results.” However, throughout the process, there was a continuous back and forth between the phases, given the iterative nature of the analyzing process. The initial, predominantly bottom-up analysis was carried out in concert with the third author. Furthermore, the later predominantly top-down phases of the analyses were reviewed with the second author.

For the second part of our study, we made use of narrative analysis to gain insight into the stories participants construct regarding their experiences. In contrast with other qualitative methods, narrative analysis focuses on the narrated account as a whole. This entails the possibility of studying, among other things, structure, genre, and plots of narratives. In other words, those elements people use to construct a story about their experiences, which enables them to make sense of their experiences (Howitt, 2010; Bamberg, 2012; Kvale and Brinkmann, 2015). In our study, we used the narrative analysis to gain insight into the different steps present in the stories and on the narrative style. The analysis started with summarizing the evolution of the psychotic process for each participant. Within these summaries the presence and expression of the proposed steps – triggering event, period of confusion, voices alluding to existential questions – were examined. Alongside this, we also specified what each step entailed for each person. Subsequently, we explored the adequacy of these steps to comprehend the whole story and the need to add possible extra steps. While doing this analysis, we were able to see the different narrative styles present in the interviews. We characterized these styles using three prototypes, which form the anchor points of a continuum. Finally, each case was placed onto this continuum. The first author conducted the narrative analysis in regular discussion with the second author.

## Results

In order to enhance the comprehensibility of the results presented beneath, Table 2 gives a short overview of the psychotic experiences of each participant.

**Table 2:** Brief overview of each participant’s psychotic experiences.

Bernie	Bernie is a 34-year-old man, who suffered from multiple drug induced psychoses. During his current episode, he got involved in a corruption scandal with influential people who went poaching on a military domain. His hallucinations mainly consist of insights which give him various tasks to make sure the good will survive over the evil (the corrupt poachers). Other insights he receives handle about a girl he loves, but who is dating one of his friends. The hallucinations guarantee him that they are meant to be, that he is the king and that she will be his queen.
Drew	Drew became psychotic during his first job, where he felt treated unfairly. While he tells us that these experiences caused a feeling of paranoia, his psychosis mainly revolves around God, the philosophy of Nietzsche and a girl he is in love with. He gets revelations that he and this girl are destined to become a couple and to give birth to the “Übermensch.” However, most of the time, Drew’s hallucinations are rather vague, unclear utterings which he needs to interpret to make sense of them, for example by making use of the birds he sees outside the hospital.
Gudrun	Gudrun is an elderly woman, who has been suffering from psychoses for over 20 years. Her hallucinations consist of the voice of her cousin, living next door, who constantly insults her. Thereby the voice especially targets Gudrun’s appearance (looking good is very important for Gudrun) and casts doubt about the love of her father, by saying that Gudrun’s father loves her (the cousin) better. On the other hand, Gudrun also hears a loving voice, which she ascribes to a former co-worker. They were both in love with each other, but as he had another relationship, a relationship between the two of them was impossible.
Howard	Howard is a 57-year-old man, who believes himself to be the highest god of the universe. However, this is not a luxury position for him. Howard has been abused as a child and crossed the line himself as an adult with a 16-year-old. He hears voices which condemn him for his deeds. Furthermore, he also has telepathic contact with two notorious child abusers and murderers, with whom he identifies. He is also convinced about being able to contact every person, dead or alive, telepathically. This also entails that all the deceased can hear him. As a result, he is hypervigilant about what he says. At last, hallucinations also gave him the message that he possesses female reproductive organs.
Joe	Joe started hearing voices, which he calls whisperers, after the death of his father. The whisperers are malevolent forces which urge him to commit crimes. Joe does not give in to the whisperers, but this means he gets punished with “the defensive noise” (tinnitus) and bodily pain. Furthermore, he links the whisperers to a system of corruption (including the police being involved in the crimes of the whisperers) and mysterious murders, including the death of his father.
Kenny	Kenny suffered from several drug induced psychoses. His current episode started after he was arrested by the police. He is convinced of being raped by the police. The voice he hears makes inappropriate sexual comments, which includes comments about the pretty nurses in the hospital, as well as comments about Kenny being a homosexual and a whore. Furthermore, the voice also encourages him to steal and to break the hospital rules, while Kenny tries to stay on the right track. Despite this, Kenny is also fond of the voice, as it keeps him company and says funny things. Next to this, Kenny also hears death threats being uttered by birds.
Matts	Matts was adopted as a child and despite having a rather good relation with his adoption family, he feels what he calls “bottomless.” In search for solid ground, he immerses himself in philosophy and religion, which eventually resulted in psychosis. Central to Matts’ psychosis is his delusional preoccupation with his theories about God and the unmeasurable in general. Hallucinations are rather limited in Matts’ case and take the form of divine revelations, which he receives in a trance-like state. He experiences a connectedness with the universe, which enables him to speak in the name of God, saying and writing things he could never come up with himself.
Roger	Roger became psychotic after his divorce. In this period, he started immersing himself in spirituality, which eventually caused him to get messages from God and being able to communicate with his deceased father-in-law. In the beginning, the voices he heard were benevolent, but after a while voices started to give him punitive and dangerous orders, which he sees as a penalty for his wrongdoings. Indeed, Roger feels guilty about his divorce and about betraying a former business partner. Roger also gets telepathic messages from a woman with whom he had a conflict. At the time of the second interview, Roger is feeling better and receives positive messages from his voices, who inspire him for new business plans.
Sophie	Sophie is a young girl, who tells a very erratic story. She is clearly overwhelmed by her voices and does not succeed in making much sense of the things she hears. Part of her hallucinations consist of voices making sexual harassing comments and accusing her of only dating her ex-boyfriend because he provided her with drugs. Hallucinations also cause Sophie to hear her friends saying things, like asking for money, while they are actually talking about something else. Another part of her hallucinations consists of senseless utterings, repeating her thoughts or the words of others. Furthermore, the voices also alter her thoughts, making her think badly about others.
Wesley	Wesley has been experiencing psychoses for twenty years. He suffers from coercive voices, which forced him to wander around for a long time and still prohibit him a lot of things, like listening to music. Wesley denotes this as “the simplification.” The voices also check upon his thoughts and his body is controlled by another patient with whom he has a telepathic connection. Furthermore, the voices inform him about the functioning of the world, which entails that humanity is actually a community of zombies which is controlled by a system of transcendental communication. Wesley hears some benevolent voices too, that give him advice to handle his illness.

### Thematic Analysis

The thematic analysis of our interviews yielded 236 episodes referring to content of hallucinations. These consisted of a broad range of voice related enunciations, going from direct testimonies of what voices say and interpretations of voices to delusional elaborations on voices and related life experiences.

We narrowed this down to the first two categories, i.e., hallucinations themselves and direct comments on hallucinations, together yielding 164 references. In Table 3 and the results below, we merge these categories to focus on the themes themselves instead of on how they came up in the data. The other two categories (delusional elaborations and related life events) helped to interpret the first two categories and were of special relevance for the later narrative analyses. However, they are not discussed in further detail, as they do not readily apply to our research question.

**Table 3:** Overview of number of times a theme comes up in the narrative of each participant.

Theme/participant	Bernie	Drew	Gudrun	Howard	Joe	Kenny	Matts	Roger	Sophie	Wesley
Parenthood and authority	7	3	3	1	7	5	6	2	/	5
Sexuality and relationships	4	7	2	8	/	13	/	14	5	/
Gender identity	/	/	6	2	/	/	/	/	/	/
Life in the light of death	1	3	/	3	3	2	/	6	/	1
What does the other want?	5	/	4	/	/	1	/	3	2	2
Unclassified content	/	/	5	1	1	8	/	7	5	1

The themes we derived from Lacanian theory, as also proposed in the method section – *gender identity, life in the light of death, parenthood and authority, sexuality, and relationships, what does the other want?* and a category for unclassified content – could all be discerned in the data. The themes regarding existential questions account for a total of 136 references; the unclassified content category consists of 28 references. Table 3 indicates how often each participant mentioned each theme. As such this table gives an impression of the scattering of themes across the participants. Whereas prevalence of a theme bears a relation with its significance, counts should not be seen as direct measures of significance, but rather as a raw indication of it. We also do not report total numbers per theme. This has little informational value, due to the differences in length and number of the interviews with each participant. As participants from time to time come back to the same event, this leads to a higher number of references in longer interviews, without always resulting in extra information.

#### Parenthood and Authority

The first theme concerns hallucinations with respect to parenthood and authority. This implies that voices either contain direct utterances by authority figures or are understood as a message coming from an authority figure. Some participants mainly heard voices specifically related to their father (Gudrun, Howard, and Matts). Others had hallucinations concerning other authority figures (Bernie, Joe, and Kenny). Moreover, for some participants, such hallucinations confirmed the active presence of a reassuring father figure (Matts in particular), whereas for others voices undermined trust in “common” authority. Voices can then come to act as an alternative authority (Drew, Kenny, Joe, Roger, and Wesley). For some, this alternative authority is especially helpful (Drew), for others it can punish or forgive misdeeds (Roger). However, this alternative authority can also take the form of an absurd power instance, subjecting participants to senseless rules (Wesley in particular).

In Matts’s case, hallucinations take the form of divine revelations that have a reassuring effect, like the neologism “*aya e emo,”* which he translates as “*verse is emotion.”* These hallucinations especially came to the fore at times when he spent discussing the existence of God at online scientific forums. Matts indicates that faith in God is vital. He was adopted as a young boy which, as he declares, entailed a lack of basic trust. Therefore, he started searching for a foot hold in philosophy and religion, which is how he found God. However, on specific online forums, he was not taken seriously, based on authority arguments nota bene: he had no scientific education and thus no right to speak, let alone question the agreed upon scientific paradigms. When his personal capacities are assailed, voices speak and restore personal trust: divine revelations confirm that God is a father figure he can trust.

Yet, for most participants, voices seemed to undermine trust in authority figures. For example, despite a good day-to-day relationship with their father, Gudrun and Howard hear voices that question their fathers love for them. Bernie, Joe, and Kenny are confronted with injustice and corruption. They hear voices that articulate a fundamental distrust in authority and rather communicate the evils of power relationships. Kenny and Joe hear voices that prompt them to “sin” and break rules.

In the case of Joe, in particular, voices are communicated by power figures from whom he cannot withdraw. He distrusts common sense authority, like the police, but is subjected to alternative authority figures. Specifically, Joe is under the spell of voices (“whisperers”) that urge him to do things which he believes are actually bad. He tries not to give in but is heavily punished for that: *“the moment you join your whisperers you become a psychopath. And it means that you will commit crimes to please those people whispering to you. To keep those devils from your body, you are compelled to participate in something. It can range from encouraging other people to start using drugs, to selling drugs or all kinds of stuff that is not allowed or that is not good for anyone.”*

Wesley is also familiar with being subjected to an evil power system. However, the commands of his voices take on very absurd forms, like being forbidden to listen to certain kinds of music. Finally, the voices of Drew and Roger also bear witness to an alternative authority system. However, in their case, messages of voices are much more sensible. Drew’s voices provide guidance in the chaos of the psychosis. They, for example, reveal to him that one of his books is the new Bible. Roger’s voices, in turn, offer reassurance or punishment for what he believes to be his wrongdoings.

#### Sexuality and Relationships

The second theme we discerned consists of hallucinations with respect to sexuality and romantic relationships. Within this theme, we classified voices explicitly uttering sexual and relational content, but also hallucinations attributed to influential persons in this area (romantic partners, sexual offenders). With regard to sexual content, voices make very explicit comments about sexuality. This is experienced as harassing (Sophie in particular) or as absurd, ridiculous, and annoying (Kenny). Others (Howard) hear voices which condemn sexual practices. Regarding relationships, messages are more positive, but always indicate a wish for an impossible relationship (Bernie, Drew, and Gudrun). Upon breakups several participants testified about voices addressing feelings of guilt (Roger and Sophie).

Bernie, Drew, and Gudrun are all in love with an inaccessible person. Within their hallucinations however, a relationship is possible. For Gudrun, this takes the form of getting affectionate advice via a telepathic connection. While Bernie and Drew in turn are offered revelations according to which they and their prospective partner are made for loving each other. Whereas for Bernie coming together is situated in a vague future, Drew’s hallucinations bring the girl he loves almost literally within reach: “*I went to bed and I was in love with a girl at that moment, the girl next-door and that girl was in a relationship with a very good friend of mine, but I heard the two of them at night, when I was sleeping or rather half asleep, half awake, I heard the two of them outside, outside on the road having a quarrel about me. And she, Leila, that girl, wanted to break-up with Simon because of me. And I acted upon that, I sent Leila flowers the next day.”*

While resulting from lack, the hallucinations of Bernie, Drew, and Gudrun are rather positive. However, this is not the case for everyone. Both Roger and Sophie speak about voices addressing feelings of guilt following a breakup. Guilt is also pivotal to Howard’s hallucinations, but in his case is situated in the field of sexuality. Indeed, Howard reports telepathic connections with two notorious child abusers and murderers. He identifies with them, as he sees himself as a child abuser too, due to the sexual relationship he once had with the 16-year-old nephew of his ex-wife. In his hallucinations, these child abusers express their guilt about their deeds. Furthermore, he also hears judging voices, which make it seem that hallucinations voice feelings of guilt he cannot express himself.

Sexuality is also a challenging phenomenon for Kenny and Sophie. They both hear voices that make sexually inappropriate or even harassing comments, as Sophie attests to here: “*Yeah, it is often, often about sex and then… bad stuff about sex like, yeah… like … guys who then say to me, for example, in a minute I’ll penetrate, or … stuff like that.*” Such voice content shocks the recipient because of its explicit nature.

#### Gender Identity

The third theme we discerned in the data is gender identity. As is seen in Table 3, only few comments could be classified under this header; however, it is very prominent in the accounts of both Gudrun and Howard. They are both confronted with voices questioning their female/male identity.

Gudrun hears the voice of a cousin living next door, commenting on her appearance: “*Yeah, well, I can tell you how she started, ‘I, euh, I think I’m a lot prettier than you are,’ that’s how she started.”* These utterings are part of Gudrun’s overall preoccupation with feminine appearance. Whereas taking good care of herself has always been important to Gudrun, it really became an issue after being rejected by a man who seemed to love her but refused to end his current relationship (see also section “*Sexuality and Relationships*”). Even years later, she cannot forget about him and is still puzzled about why she was rejected. Although she cannot embrace this issue consciously, it returns in the voices she hears, which stress the importance of looks.

Howard, on the other hand, faces concerns about femininity and masculinity: *“According to the information that I received telepathically, I should possess female sex organs.”* Being a man, which he equals to being sexually potent, has always been important to him. Howard was born with genital malformation, for which he needed multiple surgeries during childhood. He says that this made him feel inferior and created a fear of never finding a wife or having children. Being divorced and childless nowadays, the question of gender identity is addressed frequently in hallucinations.

#### Life in the Light of Death

The next theme pertains to hallucinations concerning life in the light of death. This entails participants testifying about how psychotic experiences affected and questioned their willingness to live (Bernie, Kenny, and Roger), but also about how voices gave life purpose again (Roger). Some participants say that they do not fear death, as hallucinations taught them about an afterlife (Drew, Howard, and Roger). Other participants indicate that voices help them deal with the question of to how one should live life in the world they are confronted with (Joe, Wesley).

Roger has been suffering from psychotic episodes for almost a decade. Sometimes hearing voices dampens his spirits and make him wonder why he should live. Conversely, voices also give his life a renewed purpose, and might have an encouraging effect: *“I had no more drive, didn’t know what I was doing on Earth and that continued for years and years. This year it started to change, right after my hospitalization, it started to change completely.* ***So, hmm, you mean your last hospitalization?*** *Yes, yes.* ***Yes, in what sense?*** *Yeah, in what sense euhm… I guess I came back home at the beginning of March. I’ve only been in hospital for three days I think, won’t be much longer. I had a few hard days, during which the voices left me here on the couch and let me do things I don’t even dare to talk about. Euhm, but a few days later it began to clear up. And then I slowly started to hear positive voices, positive messages.”*^1^ Thus considered, voice-hearing actively modulates how Roger feels about the purpose of life.

The wish to live is for Bernie and Kenny also heavily impacted by psychotic experiences. Kenny’s hallucinations take the form of death threats: “*Cause also, I can hear a crow eh, a crow or a raven or something like that, like ‘aargh, aargh’* [imitation of crow], *it is calling death, you know. Yeah ‘It won’t take long, boy, I will get you.’”* However, psychotic experiences might also stimulate the will to live and eliminate the fear of death, as can be found in the accounts of Drew, Howard, and Roger. They are highly convinced about the existence of an afterlife and Howard is actively in touch with the deceased: *“But yeah, I know that people in heaven can hear me. So, I need to be a bit careful with what I say, because I also have contact will all people in heaven.”*

Joe and Wesley are not so much concerned about the afterlife and more focused on the world they momentarily live in, where death is also an issue. Wesley says that humanity is a community of zombies, monitored by a transcendental system. Joe’s world is full of “whisperers” who commit mysterious murders. Both participants built these beliefs starting from information received through voices they hear. Even though, the reality they live in is rather threatening, they are relieved to know how the world works instead of being as ignorant as they were before.

#### What Does the Other Want?

The fifth existential theme we discerned concerns the question “*what does the other want?.*” Under this theme, we classified hallucinations that confront participants with an intruding other who seems to want something from them, while it remains unclear what this other actually wants. This can be rudimentary, like in the case of participants perplexed by unidentified voices that clearly seem to address them. These participants appear puzzled about why they are being addressed (Sophie and Wesley). However, even participants aware of who it is addressing them, might still be concerned about what this other is aiming for (Gudrun). For others, it is very clear that they are at the center of something. Yet, a rationale of what is happening remains lacking, which leaves them confronted with a vague enigma (Bernie and Roger). Remarkably, in response to such ungraspable, demanding others, some participants hear voices that encourage them not to trust others (Kenny) or distort thoughts about others toward negative ones (Sophie).

Gudrun is faced with the tormenting voice of her cousin who continuously offends her. As they used to get along quiet well, she is very confused as to why her cousin now acts this way. Confusion about the intention of others is also most apparent in the story of Sophie and Wesley. However, they have no clue at all about the identity of the voices addressing them, which is rather exceptional in comparison to the other participants who all ascribe clear identities to their voices (God, the devil, Obama, ex-father-in-law…). For them, the lack of identifiability puzzles them, which is especially clear in the testimony of Wesley: *“And there was also a lady who said, ‘normally I tap-dance’ and I really thought, I can’t, I can’t help you.”*

Bernie and Roger also face an obscure demanding other. However, for them there is more certainty: an immixing other disturbs their life. For Roger, this experience is rather negative, as hearing voices installs the idea of being persecuted. An air of ambiguity marks this experience, but nonetheless he does not doubt that he is the target of his voices. Bernie, however, has much more positive experiences. He believes he is on a mission. Despite the vague character of it, he is convinced about his crucial role in the world: “*Those biddings started to come and ‘you have to ensure that the good wins, cause … the bad is on the winning hand’ and you have to yeah, a bit like a pawn eh. And getting a feeling like ‘the only reason I was put on this world is to, euh, stay wandering around between all those people’ and that was actually my purpose eh. It makes you think, well, I didn’t know who, I didn’t know for sure who I was. But you do think you’re someone special, you think that at that moment.”*

#### Unclassified Content

The last category withheld from the data consists of remaining data-fragments that did not fit into one of the existential themes. Characteristic of these hallucinations is that they point to meaningful contents but not really elaborated. Contents were very vague (e.g., memories of previous experiences) or most limited (e.g., hearing one’s name), and above all fragmented in nature. As a result, content did not provide a useful starting point for further discussion of these data-fragments. However, due to the vague content, form-based characteristics played a more prominent role in the experience of these voices. Therefore, we decided to use these form-based characteristics to further subdivide the data-fragments within this theme. Half of the data-fragments in this category merely bear witness to formal disturbances. A loss of sense of agency was the most prevalent (Gudrun, Joe, Kenny, Roger, and Sophie), and one participant (Sophie) also testifies about other formal disturbances. Some participants experienced a disintegrating self via hallucinations (Kenny and Roger). Finally, voices also echoed brief comments on life in general.

Gudrun, Joe, Kenny, Roger, and Sophie all hear voices that bear testimony of a loss of sense of agency. For some, memories get an externalized quality, for example for Roger, who hears the voice of his ex-father-in-law telling him stuff he knew, but forgot about himself, like recollections of a trip to the seaside they once made together. For others, hallucinations give voice to thoughts concerning momentary events, whereby these are no longer experienced as one’s own thoughts. Joe, for example, hears a voice urging him to get up, after he was hit by a car while riding his bike. For Gudrun, at last, internal dialog becomes externalized, whereby the one hallucinated voice accuses her of saying the things she experiences as coming from another hallucinated voice.

Next, Sophie also experiences other kinds of formal disturbances. Often thoughts echo inside her head, but sometimes she also has the idea that others can hear her thoughts. This is also addressed in one of her hallucinations: *“And then he said, ‘It’s fun, isn’t it, that they can hear everything?’ … Marc was as it were telling me, but he wasn’t really saying anything like that.”* Frequently, she has the idea that the voices she hears are coming from her friends, while her friends are saying something else or are not saying anything at all.

Kenny and Roger hear voices that express feelings of disintegration. In the case of Kenny, the voice is like a bad, split-off part of the self. Kenny continuously struggles with which part is in charge, but in the meantime is also attached to his voice, as it offers company and says funny things. Roger, on the other hand, experiences a disintegration of his body image. When a voice urges him to look at his reflection, he cannot longer see himself: *“One moment, the voice in my head said to me ‘go and sit up’. I sat up, I sat up and saw my reflection. And then the same voice said, ‘go and look again’. And I didn’t see a reflection anymore, but I saw blood flowing everywhere.”*

Sometimes hallucinated messages are very short, which makes it difficult to further classify their contents. Roger heard a voice calling his name, early in the morning, when he was only just awake. For Howard, hearing this short hallucination happened in the context of megalomaniac ideas, however the hallucination didn’t add any meaning to the experience: *“‘I’m the highest God in the universe,’ I shouted. And I heard someone say, ‘even that’ and then I was God.”* Others heard brief comments on daily activities (e.g., comments during lunch) or on their condition (e.g., comments on how to cope with illness).

### Narrative Analysis

The second part of our study focuses on the narrative organization of participants’ stories concerning hallucinations. At first, we identified the different steps present in the stories. According to our hypothesis, three steps (triggering event, period of confusion, AVHs alluding on existential questions) were to expect. However, our analysis revealed that an additional step is necessary to come to a full understanding of the stories about hallucinations. This extra step has to do with disturbing experiences in the past that have a link with existential questions and constitutes the first step of the sequence. The four steps can be found in the stories of half of the participants, Bernie, Gudrun, Joe, Matts, and Roger. In the stories of the others, one or two steps were missing. Due to the addition of the extra step, we were able to make a comprehensible link between life history and content of hallucinations. Recall that according to the hypothesis, the questions hallucinations allude to, would be ones that upon confrontation trigger the psychotic episode. However, this link was not found in all stories. Nonetheless, when such a link was absent, the content of hallucinations could in all but one case (Wesley) be linked to past experiences. Thus, existential themes in hallucinations had, for all participants but Wesley, a link with experiences that caused troubles regarding existential themes, be it as a direct trigger of the psychotic episode or as an issue further away in the past. Wesley already suffered from psychosis for more than twenty years, it might be that the narrative of his hallucinations changed through the years and evolved focusing on concerns in daily life. His hallucinations are specifically linked to negative symptoms.

**FIGURE 1:**
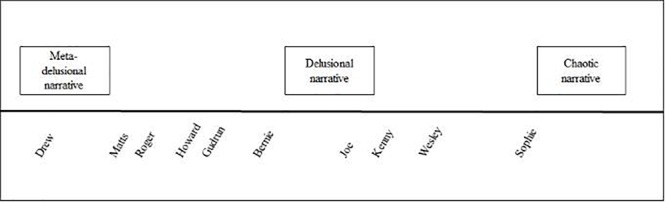
Continuum of narrative styles.

Apart of this overarching sequence of events, we observed three types of storytelling in the participants’ accounts, which we classified as meta-delusional, delusional, and chaotic narrative types. These are prototypes that constitute the anchoring points of a continuum. Figure 1 displays how we positioned each participant on this continuum. Most participants do not match perfectly with one prototype, but can be situated between prototypes, with the distance from the prototypes indicating to which one a story resembles the most.

In the following section, we clarify each narrative type with an example, indicating what each step of the hallucinatory construction entails for that participant and how these steps are imbedded in the narrative.

#### The Meta-Delusional Narrative Type

The first narrative type we discerned is a meta-delusional one. Typical for this narrative type is that participants were able to give a clear account of hallucinatory experiences, whereby they succeeded in distancing themselves from these experiences. For example, whereas Roger’s account contains some delusional elements, he can provide a structured and coherent story. The four steps of the hallucinatory logic are present and typical for the meta-delusional narrative which was clearly discernable in his story. In the narrative of Roger, we can find them as follows: Roger is an elderly man, who got divorced about a decade ago (step 1). For him, this was a very disturbing experience, which touched upon the question of how to position himself within a romantic relationship: *“So I had a divorce in that period and I had difficulties accepting that I would ever do that. Yes, like it’s totally unacceptable, you were not raised like that…”* Since his divorce he had multiple psychotic breaks. The trigger of his current psychotic episode can be situated within a conflict with a woman for whom he had feelings, but who did not appreciate him (step 2). Her attitude puzzles him and he is again confronted with the issue of how to relate to a (possible) romantic partner. What follows, is a period of confusion (step 3): *“And that has led to, a few weeks later... At first, I was utterly baffled, because here in my throat, uh, in my esophagus, there was like an accumulation of mucus… uh… and after two months someone has enlightened me that it was actually an inflammation.”* A bit later he starts to hear voices, which he attributes to that woman (step 4): *“And at once I started to receive words. Oh, it’s in English, that’s weird. And then I started euh started to reflect, asking for inspiration. Euhm, yes, and apparently it came from someone with whom I had a conflict. Telepathically transferred.”* Apart from telepathic contact with her, voices concerned feelings of guilt regarding his divorce, as already mentioned above. Romantic relationships are such a big issue in Roger’s life, that he even developed a theory to understand why relationships always fail. He relies on the idea of being on the same wavelength, albeit in a very literal way: “*And that universal law, it’s not a human law, you know, it, it entails that everything not vibrating on the same wavelength, falls apart. And we are not just talking about a box standing against some wood, but also about relationships. It just cracks, it falls apart. And I realize, the last two years, that when I get involved with a woman for whom I have feelings and she has feelings for me, it falls apart within the shortest time. Just because we aren’t, because we aren’t on the same wavelength.”*

Within these meta-delusional narratives, participants actively link voices addressing existential themes to challenging life-experiences. As such, voices become etched in a broader narrative about their lives and their overwhelming impact diminishes. Indeed, by aligning hallucinations with their personal narrative, voice content becomes in keeping with topics that also consciously occupy participants’ minds.

#### The Delusional Narrative Type

The second narrative type we discerned is the delusional narrative type. Within this type of narrative, participants narrate about hallucinatory experiences starting from a delusional point of view. Narratives are harder for an outsider to follow and as a result the four steps of the hallucinatory evolution are less discernable here. The case of Kenny is illustrative for this type of narrative. While it also contains elements of a chaotic narrative type (his narrative is rather erratic at some points) and at other points shows insight into his experiences, an essential part of Kenny’s narrative revolves around his paranoid relationship with the police. As such, Kenny’s case makes a pretty good example of how the trigger of a psychotic episode might retrospectively become part of a delusional narrative. Within Kenny’s story a detailed account about step 1 is missing. He barely speaks about his past, so no event with a distinct impact on his later hallucinations could be found. Overall, linkages with past experiences are less clear within the delusional narrative type. However, step 2, the triggering event, is highly prominent in Kenny’s story. Indeed, Kenny’s current psychotic episode started after being arrested by the police for causing nuisance, which had a huge impact on him and became the core of his delusions. He re-counts that he was drugged by the police, after which he was taken to a deserted parking spot, where he was raped and abused, before being taken to the police station. This event confuses him (step 3), bringing questions about authority to the fore: *“The police stands for justice and following the law, doesn’t it? It does not state that someone who is arrested can be raped and has no rights to water and food and things like that.”* After this arrest, Kenny’s hallucinations returned (step 4) – Kenny had psychotic episodes before but had stabilized for a while before his arrest. These voices make sexual comments: *“why don’t you show them you are a whore,” and stuff like that or “shouldn’t you give him a blowjob?”* Moreover, he hears a voice that tries to persuade him to break the law and hospital rules. We qualify Kenny’s story as delusional since an abusing outer force (the abusing police) is viewed as the source of his problems and the return of his hallucinations. While Kenny can take a certain distance of his hallucinations and he describes his experiences as a distortion of his reality, he nonetheless remains convinced about the abuse of the police. Here we see the typical psychotic double bookkeeping (Sass, 2014). So, while a narrative has been developed, it lacks the soothing quality of the meta-delusional one, as it is not integrated with the larger life story. As a result, hallucinations are very intruding and attributed to an unpredictable and manipulating outer force.

#### The Chaotic Narrative Type

The last kind of narrative we observed is the chaotic type. Characteristic of it is that participants hardly succeeded in creating a story about their hallucinatory experiences. This is very apparent in the interview with Sophie, which is rather short as she answers questions with only a few words or a single sentence. A narrative construction about her experiences is lacking. As a result, the different steps of the hallucinatory logic are hard to discern. Sophie discloses that she started dating her ex-boyfriend out of feelings of guilt. Although Sophie links this relationship and the break-up to her psychosis, we cannot conclude from her story whether this can be considered as step 1 or step 2. Nonetheless, this event introduces the question of how to position herself within a romantic relationship. Up till the moment of the interview, Sophie is really confused about everything happening to her (step 3). Unlike in a meta-delusional account, she does not clearly attribute hallucinations to challenging life events, and unlike in the delusional narrative type, she does not link these to a story about strange manipulations coming from without. Most voices she hears are hardly embedded in a narrative, which has an overwhelming impact: *“Then I started to freak out because everyone was talking to me and suddenly, I hear in the music someone with the same voice repeating everything within the music. And I totally freaked out, like I’m going crazy…”* Yet, some of the hallucinations are imbedded within a minimal narrative. She describes these as expressions of her own consciousness and they concern feelings of guilt toward her ex-boyfriend (step 4). However, for most of her hallucinations, a clear attribution is missing which is why she describes these as completely senseless utterings: *“Then I say, ‘well okay’ and then it is in my head ‘well okay, well okay, well okay, well okay.”’* Furthermore, she also testifies about hallucinatory experiences that point toward a general disturbed relationship with others: *“I noticed it also in my head, the thoughts were worse… they become worse.* ***Oh, can you tell me about it?*** *Yeah… What do I have to say? It is so much… About how I think about people and things like that. It’s all completely changing.”* Some of her hallucinations can be viewed as expressions of existential issues as well, like the sexually harassing comments mentioned in the thematic analysis section; however, she does not narratively link these to life experiences or delusional manipulations. Sophie’s account can thus be qualified as chaotic. Several elements come up but they do not blend into a narrative. She cannot make sense of her experiences, and, as a result, is very overwhelmed by everything happening to her.

## Discussion

By means of thematic and narrative analysis, this paper aimed at expanding our understanding of the content of hallucinations. Although AVHs have been researched widely, the focus is usually on formal aspects: hallucinations are conceptualized as problems in source monitoring or as signs of a disordered self-experience (Waters et al., 2012; Henriksen et al., 2015). Hitherto, only a limited amount of research has addressed the issue of AVH content and mainly explored how hallucinatory content is expressed (i.e., criticism, accusations, advice etc.; McCarthy-Jones et al., 2014), without addressing the topics articulated by voices. Indeed, little literature can be found which addresses the thematics of voice content. By making use of Lacanian theory, we aimed at mapping existential themes in hallucinations, with the assumption that hallucinations allude to existential questions. Moreover, we addressed the broader narrative within which participants embed accounts about hallucinations and examined if and how hallucinations are linked to life experiences that confronted participants with existential issues.

Our thematic analysis summarized the themes apparent in the stories participants recounted about their hallucinations. The biggest part of coded data fragments (136 out of 164) fitted into one of the existential themes, i.e., *parenthood and authority, sexuality and relationships, gender identity, life in the light of death*, and *what does the other want?* These themes, we derived from Lacanian theory (Lacan, 1959; Vanheule, 2011, 2017), are thus able to account for an important part of voice content. From this, we might conclude that voice content is most of the time not neutral or random, but really bears significance. However, existential themes are not sufficient to grasp all voice content, as we were unable to classify 28 data fragments into one of these themes. We clustered these remaining data fragments under the header *unclassified content*. As most of these fragments only showed vague or little elaborated voice-content, content-based interpretation was not possible. Instead, we further subdivided these in terms of the kind of experience they expressed. In this formal disturbances played an important role, next to experiences of a disintegrating self and comments on daily life.

Next, our narrative analysis explored links between themes addressed in hallucinations and participants’ life history. Based on Lacanian theory, we expected a narrative structure consisting of three steps, but the analysis revealed that a model with four steps better suited the stories of our participants. These four steps are: disturbing experiences in the past concerning existential questions; a triggering event addressing an existential issue; a period of confusion; and hearing voices that allude to existential questions. For nine of our 10 participants, hallucination contents could be related to an event in the past, to a triggering event, or to both. These results are in keeping with the work of authors like Karl Jaspers (McCarthy-Jones, 2012), Garety (Garety et al., 2001; Freeman and Garety, 2003), and Strand and Tidefors (2012), who also point out the link between hallucinations and personal experiences.

Furthermore, our narrative analysis mapped the ways in which participants build narratives around these building blocks. We observed three prototypical narratives about hallucinations: a meta-delusional type, a delusional type, and a chaotic narrative type. Phillips (2003) also documented different narrative constructions in the stories of psychotic patients, pointing to a fragmented self-narrative, a delusional narrative, and a sick-narrative. The first two are somewhat in line with our chaotic and delusional narrative type. We did not find a sick-narrative type, although there is some resemblance with the meta-delusional narrative type. However, Phillips focused on the narrative of the self as a whole, while our focus was on narratives concerning hallucinations specifically. Classifications of the latter are, however, missing in the literature. Indeed, general classifications of psychotic narrative types are largely lacking, which is rather surprising as narrative approaches are gaining influence in research and therapy concerning schizophrenia (e.g., Roe and Davidson, 2005; Lysaker et al., 2010). Within this framework, it is the contention that (re)building a narrative creates a coherent sense of self, which promotes recovery. Whereas it is recognized that some patients succeed better in building such a narrative than others, types of storytelling have been scarcely studied so far.

In our study, we observed links between the triggering event and the content of hallucinations. This observation, which is stressed in Lacanian theory (Lacan, 1959) as well, might suggest that hallucinations only allude to the existential question at stake in the triggering event, yet, as Table 3 shows, most participants have hallucinations that can be situated across different themes. Clearly, participants’ hallucinations had a dominant focus on a pivotal issue, which was to some extent echoed in the frequent occurrence of this theme. The dominant theme was often also the issue for which the clearest link with past experiences was apparent. Yet, while a certain event might trigger a psychotic episode and affect the content of hallucinations, this content is not limited to the question evoked by the triggering event. Indeed, when psychosis breaks through, the entire field of existential questioning seems destabilized.

Overall, this study demonstrates that hallucinations have a thematic and narrative structure that can be easily overlooked. Thematically, voices seem to address existential issues, meaning that these are not reflecting random contents. Furthermore, hallucinations are narratively organized as they in different degrees, fit into the stories participants tell about their broader life context and personal experiences. By making use of a theoretical framework, we were able to get sight of this organization. Whereas other theories might lead to other organizations and interpretations, Lacanian theory proved to be a useful framework to approach this research. Indeed, the theory enabled us to get hold of the content expressed in hallucinated voices and helped discern how hallucinations are imbedded in the stories people construct about their experiences and how these can be linked to life experiences. Obviously, characterizations earlier research developed to describe voices, i.e., criticizing, insulting, advising, etc. (Beavan and Read, 2010; McCarthy-Jones et al., 2014), also apply to the hallucinations our participants testify about. However, our study shows that analyzing content of voices on a deeper level is possible and as such it is possible to specify the topics addressed by, for example a criticizing, voice.

Within this study, our primary focus was on the content of hallucinations. However, as the unclassified content category made clear, it is difficult to study content without taking the formal aspects into account. The other way around, approaches discussing formal disturbances also cannot leave content completely aside. Indeed, in describing case examples which illustrate, for example, the sense of a disordered self, at least a minimum of content is present (see, for example, Henriksen and Parnas, 2012: detachment of the world is illustrated by a patient who describes herself as an extraterrestrial.) Moreover, as Stanghellini and Rosfort (2015) point out, there is no self-evident one-on-one relationship between implicit, formal disturbances of selfhood and the manifestation of explicit symptoms. Indeed, personal interpretation plays an important intermediate role between these two. However, the general conception of schizophrenia as a neurobiological disease makes it that few are willing to accept a possible role for personal drives in the manifestation of psychotic symptoms (Gipps and de Haan, 2018). Nonetheless, Gipps and de Haan point to the fact that, in many case examples, emotional conflict seems to play a role in psychotic experiences. Furthermore, hallucinations about the world as a community of zombies, for example, can quite easily be understood as being a metaphoric interpretation of feeling detached from the world. However, hallucinations alluding to, for example, sexuality, relationships and gender (see also Jones et al., 2018) are harder to account for from within a form-focused paradigm. Further research would thus benefit from trying to integrate both form and content in models about hallucinations and psychotic experiences in general.

Although we focused in our study on how Lacanian theory can give insight in content of hallucinations, the theory already gives an integrated account of both content and form. Indeed, according to Lacanian theory, hallucinations appear when a subject is faced with existential questions to which he cannot position himself. At such moments, language starts to act autonomously: instead of finding an answer, the subject faces a void, lacking the tools to handle this question. Upon this confrontation, unanticipated elements show up, which provide an alternative answer/reaction to these questions. As these elements are unanticipated, the subject experiences them as hallucinations: they do not feel as if they belong to himself. However, in the meantime, they define the subject with respect to the question at stake. These elements thus get nonetheless a significant meaning for the subject (Vanheule, 2018). As such, Lacanian theory gives a nice example of the interplay between content and formal disturbances. However, integration between content and form should also be possible starting from other frameworks, for example, by following the suggestions made by Stanghellini and Rosfort (2015).

Another, interesting track for future research is to replicate this research with a different population. Indeed, as cross-cultural research points out, culture has a profound impact on the experience of hallucinations. According to Luhrmann, hallucinatory experiences are shaped by what we deem plausible, based on our cultural background. People in the West are, for instance, more likely to hallucinate about God and Jesus, whereas hallucinations ascribed to spirits and ancestors are much more common in African cultures. (Luhrmann, 2011; Larøi et al., 2014; Luhrmann et al., 2015a,b) This gives rise to the question whether culture also impacts the themes that come up in voices. Although not explicitly stated, Lacanian theory leads to assume that the existential questions are universal (Vanheule, 2011). We might thus expect that similar themes will come up in a cross-cultural replication. On the other hand, cultural influence on expression and prominence of these themes seems likely. Indeed, Luhrmann et al. (2015a,b) found that Indians are much more likely to attribute voices to their kin, which may lead to a higher prominence of the *parenthood and authority* theme. Likewise, other themes might be less prominent in other cultures or it might be that other themes should be added. Furthermore, culture might also influence the narratives people construct about their hallucinations. We expect the steps of the narrative structure to be quite universal, as the linkage between hallucinatory content and life experiences was also found in former research (e.g., Garety et al., 2001; Freeman and Garety, 2003; Strand and Tidefors, 2012). However, narrative styles might be more open for cultural influence, depending on narrative traditions within a culture.

Lastly, this study is not without its limitations. The first one has to do with the interviews; as they were not conducted for our specific research question, it is possible that we missed certain information, which could have enhanced our understanding of a particular case. Furthermore, hallucinations are very overwhelming experiences, which are not expressed easily. If patients have not processed their experiences to a certain degree, building a narrative around these experiences can be difficult. Certain participants mentioned this by explicitly stating that they would or could not talk about aspects of their experiences. Undoubtedly, we missed certain contents of participants’ experiences. However, this difficulty is crucial to our understanding of psychotic experiences: if certain contents cannot be communicated, this affects the narrative construction. Indeed, as Lacan (1955–1956) suggests, the structure of psychosis is possibly expressed in such difficulties.

A further limitation concerns the quantitative data we collected. These merely count spontaneously occurring themes in interviews, and do not reflect systematic measurement. As mentioned earlier in this paper, the length of the interview was different for each participant making that the numbers are not comparable across cases. Furthermore, whereas the prevalence of a theme can give an indication of its significance, there is no strict one-on-one relationship between these two. The numbers should therefore be viewed as an illustration of the distribution of hallucinatory content across themes and participants and as of the relative prevalence of existential themes versus other voice content. However, no statistical conclusions can be made about this.

## Conclusion

In this article, we have shown the relevance of taking content into account to enhance our understanding of hallucinations. Guided by Lacanian psychoanalysis, we conducted both a thematic and a narrative analysis. Results showed that hallucinations can be understood as allusions to existential themes and are imbedded in stories participants construct about their experiences. Lacanian psychoanalysis proved to be of important pragmatic value to understand this thematical and narrative organization. This research showed that content of hallucinations is not random and can further enhance our understanding of the phenomenon. As a result, we hope that future research will also pay more attention to content and will strive to an integration of form and content-based approaches.

## Ethics Statement

This study was carried out in accordance with the recommendations of the Ethics Committee of Ghent University Hospital with written informed consent from all subjects. All subjects gave written informed consent in accordance with the Declaration of Helsinki. The protocol was approved by the Ethics Committee of Ghent University Hospital.

## Author Contributions

NM confirmed being a contributor to this work, approved it for publication, conducted both the thematic and the narrative analyses, which were audited by SV and JF, and did the original writing-up of the paper, which was then adjusted based on the feedback of both SV and JF. SV confirmed being a contributor to this work and approved it for publication. JF confirmed being a contributor to this work, approved it for publication, and conducted the interviews (one person was interviewed by SV). All authors contributed to the design of the study.

## Conflict of Interest Statement

The authors declare that the research was conducted in the absence of any commercial or financial relationships that could be construed as a potential conflict of interest.

## Footnotes

1 Text in bold refers to the words of the interviewer
